# Druggability for COVID-19: *in silico* discovery of potential drug compounds against nucleocapsid (N) protein of SARS-CoV-2

**DOI:** 10.5808/GI.2020.18.4.e43

**Published:** 2020-12-09

**Authors:** Manisha Ray, Saurav Sarkar, Surya Narayan Rath

**Affiliations:** 1 All India Institute of Medical Sciences, Bhubaneswar, Odisha 751019, India; 2 Department of Bioinformatics, Odisha University of Agriculture and Technology, Bhubaneswar, Odisha 751003, India

**Keywords:** COVID-19, molecular docking, molecular modeling, nucleocapsid protein, SARS-CoV-2

## Abstract

The coronavirus disease 2019 is a contagious disease and had caused havoc throughout the world by creating widespread mortality and morbidity. The unavailability of vaccines and proper antiviral drugs encourages the researchers to identify potential antiviral drugs to be used against the virus. The presence of RNA binding domain in the nucleocapsid (N) protein of severe acute respiratory syndrome coronavirus 2 (SARS-CoV-2) could be a potential drug target, which serves multiple critical functions during the viral life cycle, especially the viral replication. Since vaccine development might take some time, the identification of a drug compound targeting viral replication might offer a solution for treatment. The study analyzed the phylogenetic relationship of N protein sequence divergence with other 49 coronavirus species and also identified the conserved regions according to protein families through conserved domain search. Good structural binding affinities of a few natural and/or synthetic phytocompounds or drugs against N protein were determined using the molecular docking approaches. The analyzed compounds presented the higher numbers of hydrogen bonds of selected chemicals supporting the drug-ability of these compounds. Among them, the established antiviral drug glycyrrhizic acid and the phytochemical theaflavin can be considered as possible drug compounds against target N protein of SARS-CoV-2 as they showed lower binding affinities. The findings of this study might lead to the development of a drug for the SARS-CoV-2 mediated disease and offer solution to treatment of SARS-CoV-2 infection.

## Introduction

The outbreak of novel coronavirus infection has drastically affected the lives of the human population worldwide. This infection started as respiratory illness/pneumonia of unknown origin in Wuhan city of China at the end of the year 2019. The organism identified and termed as novel on 7 January 2020. The World Health Organization (WHO) declared it as a public health emergency of international concern as the disease spread to other regions of the world [1]. The official name of this infection was made as coronavirus disease 2019 (COVID-19) on 11 February 2020. The epidemic was declared a pandemic officially by WHO on 11 March 2020. The novel coronavirus is also termed, severe acute respiratory syndrome coronavirus 2 (SARS-CoV-2) [1]. SARS-CoV-2 infection mainly causes pneumonia, upper and lower respiratory tract infection with fever and cough as significant clinical symptoms. But some other symptoms include shortness of breath, muscle pain, confusion, headache, sore throat, and acute respiratory distress syndrome, leading to respiratory or multi-organ failure including renal and neurological diseases [2,3].

Coronaviruses (CoVs) are a group of large enveloped viruses with positive sense, single-stranded RNA genomes. Previously identified CoVs in human disease are the alpha CoVs (hCoV-NL63, hCoV-229E) and the beta CoVs (hCoV-0C43), severe acute respiratory syndrome CoV (SARS-CoV), and the Middle East respiratory syndrome CoV (MERS-CoV) [4]. However, among these emerging, highly pathogenic human CoVs, SARS-CoV, MERS-CoV and the newly emerged SARS-CoV-2 infection can result in life-threatening disease conditions and the potential to cause pandemic [2].

The outcome of SARS-CoV-2 sequencing (NCBI reference sequence: NC_045512.2) has proposed about the significant sequence level identity of SARS-CoV-2 with SARS-CoV (79%) rather than MERS-CoV (50%). Besides, the higher levels of transmissibility and pandemic risk of COVID-19 at an early stage has been reported in many studies [1]. In the available literatures, the size of the SARS-CoV-2 (NCBI reference sequence: NC_045512.2) genome is 30KB. The genomic virion consists of four major protein regions including matrix (M) protein, an envelope (E) protein, spike (S) protein, and a nucleocapsid (N) protein within the viral envelope [5,6]. The functional architectures of each of these viral proteins have accurately characterized. S protein primarily binds to the host cell receptor and form attachment with the host body. Alternatively, M and E proteins are involved in the formation of the viral envelope [6]. Similarly, SARS-CoV-2 protein N is a multifunctional RNA binding protein, necessary for viral RNA transcription, replication and/or assembly of virus [6]. Interestingly, a unique N-terminal RNA binding domain of SARS-CoV-2 N protein has identified as a novel antiviral drug target site [7]. The viral N protein packages the genome into long, flexible, and helical RNP complexes, called nucleocapsids which protect the SARS-CoV-2 virion structure [5]. Additionally, N protein has a significant contribution towards timely replication and reliable transmission of SARS-CoV-2 during its life cycle. Therefore, N protein (PDB ID: 6VYO) can be considered as a novel drug target of SARS-CoV-2.

The SARS-CoV-2 infection has created a dangerous pandemic situation due to its quick transmission and deadly nature. It has affected both the health and economy of human population across the globe tremendously. Many ongoing pieces of research are trying to develop vaccines to control this situation, but all are in various phases of trials. Thus, the present study has focused on *in silico* discovery of potent leads from several antiviral drugs and compounds of plant origin against SARS-CoV-2 infection. The present study would throw lights on the discovery of antiviral drug against SARS-CoV-2.

## Methods

### Sequence retrieval and construction of phylogenetic tree

Nucleocapsid protein sequences of total 49 CoV species and/or strains including SARS-CoV-2 were retrieved in FASTA format from NCBI web server (https://www.ncbi.nlm.nih.gov/) on 30 March 2020. Two N proteins of Ebola and H1N1 virus were included to study evolutionary divergence across species. Further, total 51 N protein sequences were aligned using MUSCLE algorithm of Molecular Evolutionary Genetics Analysis 7 (MEGA 7) package [8]. The resulted alignment was used to generate phylogenetic tree using neighbour joining (NJ) method of MEGA 7 for 1,000 bootstrap replicates.

### Conserved domain search

Functional domains of SARS-CoV-2 N protein (YP_009724397.2) were identified using NCBI conserved domain database (CDD) (https://www.ncbi.nlm.nih.gov/Structure/cdd/wrpsb.cgi) search. The CDD is a collection of domain models which imports information from Pfam, SMART, COG, and NCBI to provide a more accurate assessment of neighbor relationships between protein sequences [9].

### Prediction of structural element

The secondary structure of SARS-CoV-2 N protein was predicted from its complete amino acid sequence (accession No. YP_009724397.2) using PSIPRED 4.0 algorithm [10]. Similarly, protein disorder portion and membrane helix region was predicted by using DISOPRED3 [10] and MEMSAT-SVM algorithm [10] of PSIPRED web server (http://bioinf.cs.ucl.ac.uk/psipred/).

### Retrieval and preparation of 3D structure

Available N-terminal domain structure (PDB ID: 6VYO) of SARS-CoV-2 N protein was retrieved from Protein Data Bank (PDB) (https://www.rcsb.org/). Initially, hydrogen atoms were added to protein structure after removal of all water and other hetero molecules. Further, energy minimization was performed using Discovery Studio 3.5 suite to obtain a properly optimized structure of target protein.

### Drug-binding cavity prediction

In absence of knowledge on exact drug-binding site, probable binding cavity within SARS-CoV-2 N protein was predicted using metaPocket 2.0 (https://projects.biotec.tu-dresden.de/metapocket/). MetaPocket tool identifies cavities on protein surface for drug-binding site prediction using multiple computational approaches [11] such as PASS11, LIGSITE, Fpocket, SURFNET, GHECOM, and ConCavity.

### Selection of ligand molecules

Different natural compounds of plant origin reported with antiviral, anti-inflammation, anti-influenza, anti‒human immunodeficiency virus, anti-hepatic properties were shortlisted from different literatures. In addition, few Food and Drug Administration approved, and investigational antiviral drugs were also selected from Drug Bank (https://www.drugbank.ca/) database for further investigation.

### Ligand structure retrieval and correction

Three-dimensional structures of natural ligands were retrieved from PubChem (https://pubchem.ncbi.nlm.nih.gov/) database in SDF format and converted into PDB format using Discovery Studio 3.5 suite. Similarly, PDB structures of antiviral drugs were collected from the Drug Bank (https://www.drugbank.ca/). Further, structure optimization and protonation state of all ligands were achieved using Discovery Studio 3.5 suite.

### Molecular docking

Molecular docking was performed between all selected ligands (phytochemicals and antiviral drugs) and the drug target (N protein, PDB ID: 6VYO) separately in order to identify the most efficient inhibitor against SARS-CoV-2. AutoDock 4.2 (http://autodock.scripps.edu/) and AutoDock Tools 4 tool [12] were used to perform molecular docking study. The N-terminal RNA binding domain of SARS-CoV-2 N protein was observed as a homotetramer structure; therefore, only chain A of the available crystal structure was employed for docking analysis. Prior to docking, Kollman charges and polar hydrogen atoms were added to the target structure. Both ligand and receptor structures were prepared using ADT tool and converted to pdbqt format before docking. A virtual grid box was set around the drug-binding cavity of the target structure with size of 74, 78, and 74 Å in x, y, and z direction in spacing of 0.375 Å. Semi flexible docking was performed by maintaining target structure as rigid and allowing flexibility to ligand molecules within the drug-binding pocket [13]. Lamarckian genetic algorithm was used with 25,000,000 energy evaluation steps for each dock run. Auto dock generated 10 conformers based on free binding energy for each protein-ligand complex. The most energetically favorable (lowest energy) binding complex was considered for analysis. Further analysis and presentation of atomic interaction between docked complexes were performed using PyMol molecular graphics tool (http://www.pymol.org).

## Results

### Molecular phylogeny ascertained sequential divergence of SARS-CoV-2 N protein

**Fig. 1.**
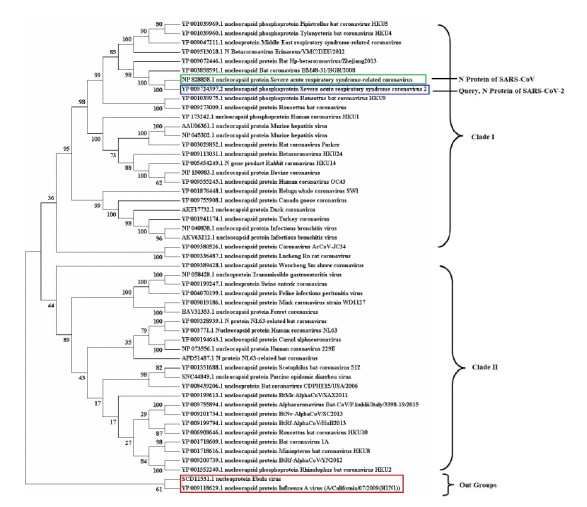
Phylogenetic tree were presented among 49 nucleocapsid (N) protein sequences of severe acute respiratory syndrome coronavirus (SARS-CoV) and SARS-CoV-2 from different species. The number in the left side of tree denotes bootstrap frequency for each taxon. The N protein of out group (Ebola and H1N1) sequences and the target SARS-CoV-2 protein were highlighted using red and blue outline respectively. Similarly, N protein sequence of SARS-CoV was highlighted using green outline.

Total 49 N proteins different CoV species, including SARS-CoV-2 (Table 1) were retrieved to construct the phylogenetic tree. Again, protein sequences of two distance homologues of SARS-CoV-2 such as Ebola (accession No. SCD11531.1) and H1N1 (accession No. YP_009118629.1) virus were included within the tree in order to establish sequential divergence pattern across species. The phylogenetic tree was constructed using NJ method [14] with tree evaluation step for 1,000 bootstrap replicates. The resulted rooted tree (Fig. 1) clustered into two major clades. Total 49 species were diversified within both of the clades (clade-I, 26; clade-II, 23). The target N protein sequence of SARS-CoV-2 (accession No. YP_009724397.2) was grouped with SARS-CoV (severe acute respiratory syndrome-related virus) (accession No. NP_828858.1) sequence within clade-I with branch frequency of 100% which pointed out regarding their significant evolutionary closeness. One separate clade was formed within the tree with branch frequency of 61% among the two outgroups (Ebola and H1N1) which clearly revealed their divergence from all other 49 sequences.

### Functional domain identified for SARS-CoV-2 N protein

**Fig. 2.**
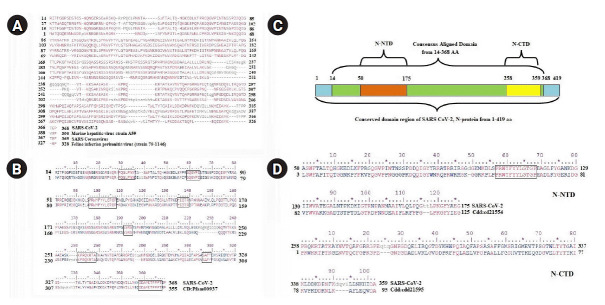
Conserved functional domains of severe acute respiratory syndrome coronavirus 2 (SARS-CoV-2) nucleocapsid protein. (A) Sequence alignment between SARS-CoV-2 and members of super family (pfam00937). (B) The alignment between SARS-CoV-2 and consensus sequence of pfam00937 nucleocapsid protein. The conserved amino acid patterns were highlighted using boxes. (C) All functional domain regions of SARS-CoV-2 nucleocapsid protein were presented in schematic diagram. N-NTD, nucleocapsid protein N-terminal domain; N-CTD, nucleocapsid protein C-terminal domain. (D) The sequence alignment of N-NTD (50-175) and N-CTD of SARS-CoV-2 with their respective conserved domain family.

The complete sequence of SARS-CoV-2 N protein (accession No. YP_009724397.2) comprises of 419 amino acids. All functional domain regions within the N protein sequence of SARS-CoV-2 were identified from its conserved pattern among the members of beta CoV nucleocapsid protein family. The conserved domains were observed within the aligned region of SARS-CoV-2 N protein from 14‒368 amino acids (Fig. 2A) with the members of the superfamily (pfam00937) (Fig. 2B). The CD search identified one. N-terminal (50‒175 amino acids) and one C-terminal (258‒359 amino acids) functional domain (Fig. 2C) with good bit score (424.07) and lowest e-value (7.05e-148). The nucleocapsid N-terminal domain (NTD) of SARS-CoV-2 was showed significant similarities with the conserved domain of family cd21554 whereas the C-terminal domain (CTD) found conserved within the family members of cd21595 (Fig. 2D).

### Structural elements of SARS-CoV-2 N protein

**Fig. 3.**
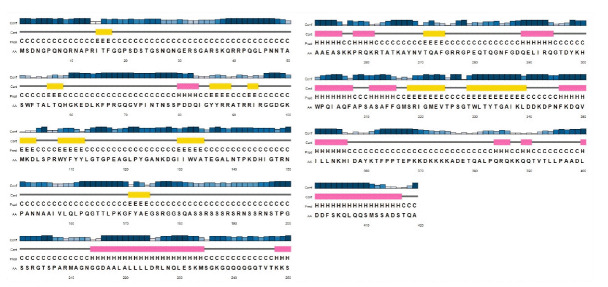
Predicted secondary structural elements for full length nucleocapsid protein of severe acute respiratory syndrome coronavirus 2. Helix, pink cylinder; Sheet, yellow cylinder.

In the absence of full-length structure, the secondary structural elements of SARS-CoV-2 N protein were predicted from its primary sequence using PSIPRED web server. Secondary structural elements such as two long, eight medium, two short helical regions and two medium, nine short β-sheets were predicted within the complete sequence of SARS-CoV-2 N protein (Fig. 3).

**Fig. 4.**
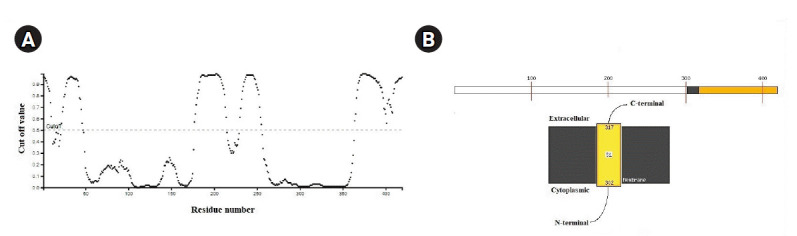
(A) The disorder plot of severe acute respiratory syndrome coronavirus 2 (SARS-CoV-2) nucleocapsid protein was deciphered. X-axis: amino acid residue number; Y-axis: disorder cut off value. Black color dots were used to plot disorder values on the Y-axis for the corresponding amino acids on X-axis. (B) Representation of sub-cellular localization of SARS-CoV-2 nucleocapsid N-terminal domain.

Most of the NTD (50‒175) regions were predicted as β-sheets and coils. On the contrary, structural elements such as helices, β-sheets, and coils were observed within CTD (258‒359) regions (Fig. 3). Further, highly disordered regions of SARS-CoV-2 N protein were observed above the cut off score (0.5) from amino acid positions 1‒50, 180‒250, and 350‒419 (Fig. 4A). However, significant disorder portions were absent within the both NTD (50‒175) and CTD (258‒359) regions (Fig. 4A). According to MEMSAT-SVM algorithm, the sub-cellular localization of SARS-CoV-2 nucleocapsid NTD was found as cytoplasmic, whereas a small C-terminal transmembrane region was noticed from 302‒317 amino acids (Fig. 4B).

### Structure preparation and active site identification of N protein NTD

**Fig. 5.**
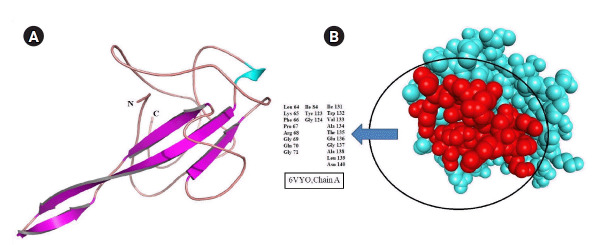
(A) Cartoon representation of severe acute respiratory syndrome coronavirus 2 (SARS-CoV-2) nucleocapsid protein (PDB ID: 6VYO, chain A) structure. Β-sheet, pink colour arrows; Coil, tube. (B) Space filling representation. Active drug-binding pocket was highlighted using red colour within the structure.

Homology search using BLASTP algorithm revealed the structure of N-terminal RNA binding domain occupied 30% region of SARS-CoV-2 N protein (accession No. YP_009724397.2) sequence with 100% identity. Therefore, the three-dimensional structure of SARS-CoV-2 N protein was retrieved and processed for structural correction and optimization. The possible drug-binding cavity of SARS-CoV-2 N protein was predicted in the absence of literary evidence. Algorithm of metaPocket was generated top three hits after clustering the results of PASS11, LIGSITE, Fpocket, SURFNET, GHECOM, and ConCavity. Out of these three, the large active pocket was considered a possible drug-binding cavity (Fig. 5).

### Structure preparation natural/synthetic ligands against SARS-CoV-2 N protein

As of literature, a total of eight natural compounds of plant origin and three synthetic compounds (Table 2) were identified with antiviral properties, therefore, prepared to dock against SARS-CoV-2 N protein.

Again, seven antiviral drugs (Table 3) were also included within the study to discover potent inhibitor against N protein of SARS-CoV-2. Finally, 3D structures of a total of eighteen ligands were extracted from online databases (PubChem/Drug Bank) and prepared for docking study.

### Molecular docking identified efficient ligand against SARS-CoV-2 N protein

**Fig. 6.**
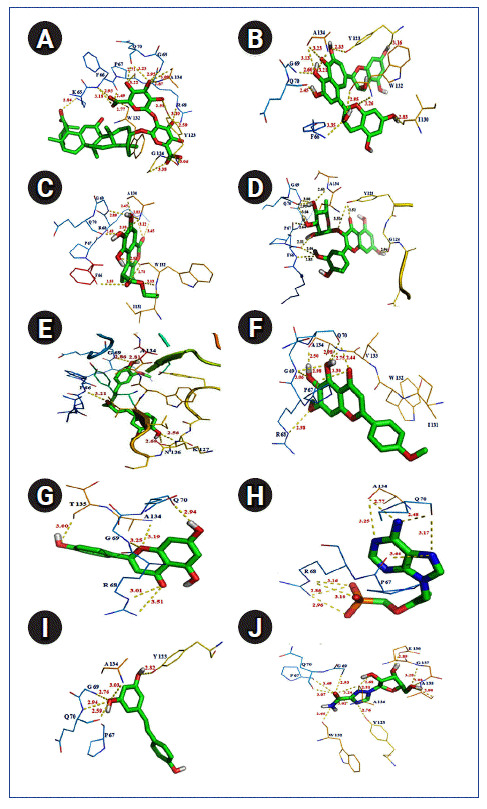
Polar interaction between severe acute respiratory syndrome coronavirus 2 (SARS-CoV-2) nucleocapsid protein with natural/synthetic compounds: glycyrrhizin (A), theaflavin (B), ethylbrevifolincarboxylate (C), quercitrin (D), curcumin (E), ladanein (F), apigenin (G), tenofovin (H), resveratrol (I), and ribavirin (J).

Molecular docking is an efficient technique to identify the binding affinity of a drug compound against a drug target [15,25]. Therefore, all possible inhibitors were docked separately against SARS-CoV-2 N protein to discover effective ligand and important atomic interaction between protein-ligand complexes within the drug-binding cavity. The resulted in free binding energy, and the inhibition constant of each binding complex was reported in Table 4. According to docking energy score and inhibition constant (KI), total eight antiviral compounds such as glycyrrhizic acid (‒12.61 kcal/mol; KI, 573.72 pm), theaflavin (‒10.35 kcal/mol; KI, 26.03 nM), diosgenin (‒10.06 kcal/mol; KI, 42.53 nM), U18666A (‒9.08 kcal/mol; KI, 219.38 nM), ethyl brevifolincarboxylate (‒9.07 kcal/mol; KI, 226.42 nM), quercitrin (‒9.04 kcal/mol; KI, 238.18 nM), curcumin (‒8.68 kcal/mol; KI, 434.59 nM), and ladanein (‒8.19 kcal/mol; KI, 988.63 nM) showed good binding efficiency than rest of the compounds (Table 4). Presence of an ample number of polar interactions has a significant contribution towards the stability of a specific ligand within the binding site of drug target. Therefore, h-bond interaction between the drug target and ligands were inspected. Interestingly, good binding affinity and strong h-bond interaction within distance ≤ 3.5 Å from binding cavity were identified in case of 10 suitable compounds such as glycyrrhizic acid (‒12.61 kcal/mol; h-bond, 16 nos), theaflavin (‒10.35 kcal/mol; h-bond, 11 nos), ethyl brevifolincarboxylate (‒9.07 kcal/mol; h-bond, 6 nos), quercitrin (‒9.04 kcal/mol; h-bond, 11 nos), curcumin (‒8.68 kcal/mol; h-bond, 5 nos), ladanein (‒8.19 kcal/mol; h-bond, 8 nos), apigenin (‒7.98 kcal/mol; h-bond, 6 nos), tenofovir (‒6.92 kcal/mol; h-bond, 9 nos), resveratrol (‒6.91 kcal/mol; h-bond, 5 nos), ribavirin (‒6.41 kcal/mol; h-bond, 12 nos), indicated about their efficacy to block the important site within the RNA binding domain of SARS-CoV-2 N protein (Tables 4 and 5, Fig. 6).

**Fig. 7.**
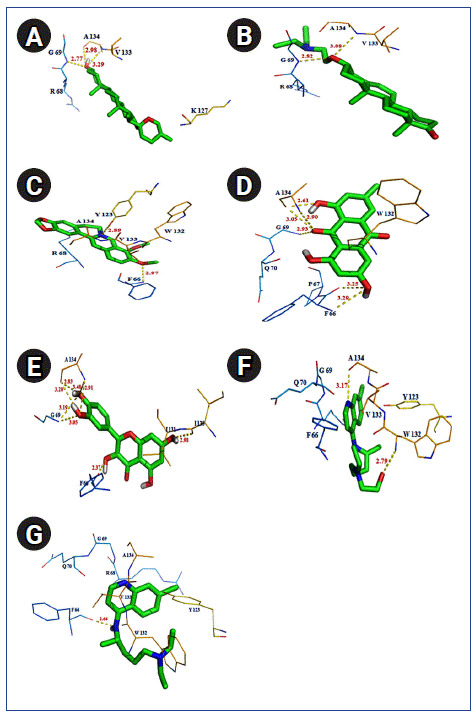
Polar interaction between severe acute respiratory syndrome coronavirus 2 (SARS-CoV-2) nucleocapsid protein with natural/synthetic compounds: diosgenin (A), U18666A (B), berberine (C), emodin (D), quercetin (E), hydroxyl chloroquine (F), and chloroquine (G).

To its support, few amino acid residues such as PHE 66, PRO 67, ARG 68, GLY 69, GLN 70, TYR 123, TRP 132, and ALA 134 were found commonly interacting with all of these ligands within the binding cavity of SARS-CoV-2 N protein. However, presence of h-bond interaction with quite good binding energy and inhibition constant values were also noticed in case of rest seven antiviral compounds such as diosgenin (‒10.06 kcal/mol; KI, 42.53 nM; h-bond, 3 nos), U18666A (‒9.08 kcal/mol; KI, 219.38 nM; h-bond, 2 nos), berberine (‒7.87 kcal/mol; KI, 1.69 µM; h-bond, 2 nos), emodin (‒7.82 kcal/mol; KI, 1.86 uM; h-bond, 6 nos), quercetin (‒7.47 kcal/mol; KI, 3.33 µM; h-bond, 8 nos), hydroxychloroquine (‒7.35 kcal/mol; KI, 4.07 µM; h-bond, 2 nos), chloroquine (‒6.86 kcal/mol; KI, 9.34 µM; h-bond, 1 nos) inbound form with SARS-CoV-2 N protein (Table 4, Fig. 7). Overall docking study confirmed the binding potential of the discussed phytochemicals and drugs, against drug target, Nucleocapsid protein of SARS-CoV-2.

## Discussion

The SARS-CoV-2 or COVID-19 pandemic has created an alarming situation due to severe infection and death rate worldwide. Researchers all over the world are in search to identify novel drug/vaccine target as well as the development of drug/vaccine to combat the disease. Several recent studies have been reported probable synthetic drug candidates such as conivaptan, amyrin, ZINC000027115482 [26], ritonavir, lopinavir, umifenovir [27], theophylline, pyrimidine [28], simeprevir and grazoprevir [29] against nucleocapsid protein of SARS-CoV-2. As, N protein has a vital role for the survival and growth of SARS-CoV-2 thus authors focused on the discovery of potential natural or synthetic compounds to block its regular mechanism. In support of the present scenario, the current study has tried to conduct some critical analyses on important drug target, i.e., nucleocapsid (N) protein of SARS-CoV-2. The present research also focuses on *in silico* discovery of potent natural/synthetic compounds against the virus.

The phylogenetic study among different CoV species community identified the close relation and less diversification between N proteins of SARS-CoV and SARS-CoV-2, which indicates the high similarities between those species. The protein family sequence similarity search or the conserved domain search points out the versatility of SARS-CoV-2 N protein, which is predicted by the conserved amino acid regions from different members CoV superfamilies such as SARS-CoV, murine CoV (murine hepatitis virus) and alpha CoV-1 species (Feline infectious peritonitis virus).

Primary sequence analysis resulted in two crucial functional domain regions both in N and C terminals of SARS-CoV-2. Interestingly, the NTD comprises RNA binding site, which signifies its importance towards a viral cellular mechanism. To its support, the available crystal structure of NTD SARS-CoV-2 N protein was retrieved and utilized in further study. The SARS-CoV-2 N protein had no binding site information including drug-binding sites till the end of March 2020, which influences the researchers to predict the drug-binding pocket in RNA binding domain of N protein. But recently, Kang et al. [30] reported about the crystal structure and showed the drug-binding pocket (including the amino acids Tyr 110, Tyr 112, Tyr 55, Ala56, and Arg89) of N protein with PDB ID 6M3M whereas this present study predicted the binding domain in SARS-CoV-2 N protein (PDB ID: 6VYO) with amino acids positioned from 64‒71, 84, 123‒124, and 131‒140. This study represents the maximum similarities between the crystal structure binding pocket and the presently identified drug-binding pocket in N protein, which should be considered while deciding a drug for trial in the treatment of the disease.

Today, the death report of COVID-19 from different corner of the globe is drastically increasing due to the absence of an effective antiviral drug. To overcome this situation, eighteen compounds, including natural compounds of plant origin and antiviral drugs, were docked into the drug-binding cavity of N protein to identify potential ligands against SARS-CoV-2. This study has been able to find the binding efficiency of a few phytochemicals (Theaflavin, curcumin, ladanein), and a few drug compounds (glycyrrhizic acid, ethyl brevifolin caboxylate, and quercitrin) against N protein of the virus. This might serve as information about their potential to be a treatment option for SARS-CoV-2. The antiviral effects of phytochemicals such as Theaflavin, curcumin, and ladanein, against many pathogenic viruses, have already been well studied and reported. Theaflavin is known to prevent from influenza virus by inhibiting its replication [15].

Similarly, curcumin has antiviral properties against H1N1 Influenza and FIPV [16]. Again, the inhibitory effect of ladanein against hepatitis C virus infection [17] is also well studied. Thus, these compounds may be useful as an anti-infective agent against COVID-19. Antiviral drugs such as glycyrrhizic acid, ethyl brevifolincarboxylate, and quercitrin have inhibitory effect against [18,23] hepatitis B and C virus. But, glycyrrhizic acid and quercetin are associated with severe side effects such as hypokalemia, oedema, rhabdomyolysis or myoglobinuria, mitochondrial toxicity, and mutagenicity [31,32]. However, according to the resulted binding affinities and the presence of H-bonds glycyrrhizic acid and theaflavin can be considered as suitable drug compounds against SARS-CoV-2 N protein. In regards to toxicity associated with glycyrrhizic acid, the use of natural compound, i.e., theaflavin may be more effective against COVID-19. Other than the mentioned natural/synthetic compounds, few others such as diosgenin [17], U18666A [19], apigenin (*Ocimum sanctum*) [20], resveratrol (*Vitis labrusca*) [21], berberine (*Berberis vulgaris*) [22], emodin (*Radix et Rhizoma Rhei, Radix Polygoni Multiflori*) [24], and tenofovir (*Phyllanthus niruri*) [18] has shown stable binding interaction with SARS-CoV-2 N protein. Hence they may also be studied for further validation.

The COVID-19 outbreak has caused havoc throughout the world, changing the course of human lives. Researchers are trying to design a vaccine against SARS-CoV-2 but that might take some time. This study attempts to find a drug for treating the disease condition, which will help to save human lives and mitigate the sufferings of millions of people infected by the virus worldwide. Some antivirals phytocompounds and synthetic drugs have been analyzed in this *in silico* study, which would target the N protein, responsible for replication of SARS-CoV-2 in the host body. Of all the compounds in this study, glycyrrhizic acid and theaflavin can be used as the antiviral drug, as they showed a higher binding affinity with the target protein. The effective drug candidates would be helpful to prevent the SARS-CoV-2 viral N protein and to reduce the risk of infection in the host body.

## Figures and Tables

**Table 1.** Nucleocapsid proteins from different coronavirus species collected from NCBI

No.	Species name	NCBI accession	Length (bp)
1	Duck corornavirus (avian CoV)	AKF17732.1	414
2	Turkey coronavirus (avian CoV)	YP_001941174.1	409
3	Infectious bronchitis virus (avian CoV)	NP_040838.1	409
4	Infectious bronchitis virus (avian CoV)	AKV63212.1	409
5	Rat CoV parker (murine CoV)	YP_003029852.1	454
6	Murine hepatitis virus (murine CoV)	AAU06361.1	454
7	Murine hepatitis virus (murine CoV)	NP_045302.1	454
8	Bovine coronavirus (beta CoV)	NP_150083.1	448
9	Human coronavirus OC43 (beta CoV)	YP_009555245.1	448
10	Middle East respiratory syndrome-related coronavirus (MERS-CoV)	YP_007188585.1	411
11	Mink coronavirus 1	YP_009019186.1	376
12	Feline infectious peritonitis virus (alpha coronavirus 1)	YP_004070199.1	377
13	Transmissible gastroenteritis virus (alpha coronavirus 1)	NP_058428.1	382
14	Rousettus bat coronavirus HKU9	YP_001039975.1	468
15	Pipistrellus bat coronavirus HKU5	YP_001039969.1	427
16	Canada goose coronavirus	YP_009755908.1	414
17	Tylonycteris bat coronavirus HKU4	YP_001039960.1	423
18	Severe acute respiratory syndrome-related coronavirus (SARS-CoV)	NP_828858.1	422
19	Severe acute respiratory syndrome coronavirus-2 (SARS-CoV-2)	YP_009724397.2	419
20	Alpha coronavirus bat-CoV/P.kuhlii/Italy/3398-19/2015	YP_009755894.1	432
21	Miniopterus bat coronavirus 1	YP_001718609.1	389
22	Wencheng Sm shrew coronavirus	YP_009389428.1	366
23	Coronavirus AcCoV-JC34	YP_009380526.1	389
24	Lucheng Rn rat coronavirus	YP_009336487.1	391
25	NL63-related bat coronavirus	APD51488.1	433
26	NL63-related bat coronavirus	YP_009328939.1	407
27	Rousettus bat coronavirus	YP_009273009.1	443
28	Ferret coronavirus	BAV31353.1	374
29	BtMr-AlphaCoV/SAX2011	YP_009199613.1	429
30	BtNv-AlphaCoV/SC2013	YP_009201734.1	431
31	BtRf-AlphaCoV/HuB 2013	YP_009199794.1	383
32	BtRf-AlphaCoV/YN2012	YP_009200739.1	375
33	Swine enteric coronavirus	YP_009199247.1	382
34	Camel alpha coronavirus	YP_009194643.1	382
35	Beta coronavirus HKU24	YP_009113031.1	443
36	Bat-Hp-Betacoronavirus/Zhejiang 2013	YP_009072446.1	418
37	Betacoronavirus Erinaceus/VMC/DEU/2012	YP_009513018.1	424
38	Bat coronavirus CDPHE15/USA/2006	YP_008439206.1	425
39	Rousettus bat coronavirusV HKU10	YP_006908646.1	402
40	Rabbit coronavirus HKU14	YP_005454249.1	444
41	Beluga whale coronavirus SW1	YP_001876448.1	379
42	Miniopterus bat coronavirus HKU8	YP_001718616.1	422
43	Rhinolophus bat coronavirus HKU2	YP_001552240.1	375
44	Scotophilus bat coronavirus 512	YP_001351688.1	394
45	Human coronavirus HKU1	YP_173242.1	441
46	Human CoV NL63	YP_003771.1	377
47	Bat coronavirus BM48-31/BGR/2008	YP_003858591.1	417
48	Human coronavirus 229E	NP_073556.1	389
49	Porcine epidemic diarrhea virus	NP_598314.1	441

**Table 2.** Eleven ligand molecules (natural and synthetic) and antiviral properties

No.	Natural/Synthetic compounds	Pub Chem CID	Source/Plant name	Property	Virus	Reference
1	Theaflavin	135403798	*Camellia sinensis *(tea plant)	Prevents influenza by inhibiting replication using potentially directs virucidal effect	H1N1	[15]
2	Curcumin	969516	*Curcuma longa *L*.* (turmeric)	Antiviral activity against FIPV	FIPV, HIV, influenza	[16]
				Inhibition of HIV-1 and HIV-2 proteases		
				Inhibition of haemagglutination		
3	Diosgenin	99474	Synthetic	Effectively blocks the replication of hepatitis C virus	Hepatitis C virus	[17]
4	Ladanein	3084066	*Marrubium peregrinum *L.	Effectively inhibits the post attachment entry step of hepatitis C virus	Hepatitis C virus	[17]
5	Quercetin	5280343	*Phyllanthus niruri*	Inhibits virus replication and viral nucleocapsid formation by inhibiting DNA polymerase of hepatitis B	Hepatitis B/C virus	[17,18]
6	Ethyl brevifolincarboxylate	5487248	Synthetic	Inhibits virus replication and viral nucleocapsid formation by inhibiting DNA polymerase of hepatitis B	Hepatitis B virus	[18]
7	Quercitrin	5280459	*Phyllanthus niruri*	Inhibit virus replication and viral nucleocapsid formation by inhibiting DNA polymerase of hepatitis B	Hepatitis B virus	[18]
8	U18666A	9954082	Synthetic	Inhibits the proliferation of type 1 FIPV	Feline infectious peritonitis virus	[19]
9	Apigenin	5280443	*Ocimum sanctum *(Tulsi)	Prevents the early multiplication of H1N1 virus and control the viral growth	H1N1	[20]
10	Resveratrol	445154	*Vitis labrusca*	Effectively reduce the inflammatory cell production and pro-inflammatory cytokine accumulation	Inflammatory virus	[21]
11	Allicin	65036	*Allium sativum* (garlic)	Inhibit virus penetration and proliferation (inhibit cell proliferation, protect the heart injury, liver damage, anti-inflammation)	Influenza	[31]

CID, compound ID; FIPV, feline infectious peritonitis virus; HIV, human immunodeficiency virus.

**Table 3.** Seven antiviral drugs and medicinal value

No.	Synthetic/Natural drug compound	DB ID	Status	Source/Plant name	Treatment/Property	Reference
1	Glycyrrhizic acid (glycyrrhizin)	DB13751	Approved, experimental	*Glycyrrhiza glabra*	Inhibit viral replication of SARS-CoV	[23]
2	Ribavirin	DB00811	Approved	Synthetic	Effective against chronic hepatitis C virus, SARS-CoV	PMID:18565019, [17]
3	Tenofovir	DB14126	Experimental, investigational	*Phyllanthus niruri*	Hepatitis B virus	[18]
4	Berberine	DB04115	Approved, investigational	*Berberis vulgaris*	Prevents the HIV-PI induced inflammation	[22]
5	Emodin	DB07715	Investigational	*Radix et Rhizoma Rhei, Radix Polygoni Multiflori*	Blocks the S protein of SARS-CoV and ACE2 interaction	[24]
6	Chloroquine	DB00608	Approved, investigational, vet approved	Synthetic	HIV, influenza A/H5N1, SARS-CoV, human coronavirus 229E	PMID: 23648708
7	Hydroxy chloroquine	DB01611	Approved	Synthetic	HIV, DENV	PMID:25321315

DB ID: Drug Bank ID; SARS-CoV, severe acute respiratory syndrome coronavirus; PMID: PubMed ID; HIV, human immunodeficiency virus; ACE2, angiotensin-converting enzyme 2; DENV, Dengue virus.

**Table 4.** Docking scores of 18 ligands against SARS-CoV-2 N protein

No.	Ligands (phytochemicals/drugs)	Docking energy scores (kcal/mol)	Intermolecular energy (kcal/mol)	Inhibition constant (KI)
1	Glycyrrhizic acid (glycyrrhizin)	‒12.61	‒14.7	573.72 pM
2	Theaflavin	‒10.35	‒13.63	26.03 nM
3	Diosgenin	‒10.06	‒10.35	42.53 nM
4	U18666A	‒9.08	‒10.87	219.38 nM
5	Ethyl brevifolincarboxylate	‒9.07	‒10.86	226.42 nM
6	Quercitrin	‒9.04	‒12.02	238.18 nM
7	Curcumin	‒8.68	‒11.66	434.59 nM
8	Ladanein	‒8.19	‒9.68	988.63 nM
9	Apigenin	‒7.98	‒9.17	1.43 μM
10	Berberine	‒7.87	‒8.47	1.69 μM
11	Emodin	‒7.82	‒8.71	1.86 μM
12	Quercetin	‒7.47	‒9.26	3.33 μM
13	Hydroxy chloroquine	‒7.35	‒10.04	4.07 μM
14	Tenofovir	‒6.92	‒8.41	8.53 μM
15	Resveratrol	‒6.91	‒8.4	8.63 μM
16	Chloroquine	‒6.86	‒9.25	9.34 μM
17	Ribavirin	‒6.41	‒8.2	19.88 μM
18	Allicin	‒4.69	‒6.18	363.41 μM

SARS-CoV-2, severe acute respiratory syndrome coronavirus 2.

**Table 5.** Polar interaction (distance ≤ 3.5 Å) between selected antiviral compounds and nucleocapsid protein of SARS-CoV-2

No.	Phytochemical/Drug	H-bond residue	Bond	Length (A^0^)
1	Glycyrrhizic acid (glycyrrhizin)	LYS 65	NZ…O	2.84
		PHE 66	N…O	3.18
			N…O	2.93
			OH…O	2.49
		PRO 67	OH…O	3.22
		ARG 68	NE…O	3.10
			NH1…O	2.59
		GLY 69	N…O	2.95
		GLN 70	OH…O	2.71
			OH…O	3.23
		TYR 123	OH…O	2.91
			OH…O	3.06
		GLY 124	OH…O	3.38
		TRP 132	OH…O	2.77
		ALA 134	N…O	2.87
			OH…O	3.09
2	Theaflavin	PHE 66	N…OH53	3.35
		GLY 69	N…O5	2.60
		GLN 70	O…H60	2.45
		TYR 123	OH…O9	2.96
			OH…O9	3.16
		ILE 130	O…H64	2.83
		TRP 132	O…H53	2.95
			N…O1	3.26
		ALA 134	O…OH59	3.13
			N…OH59	3.21
			OH…O10	3.23
3	Ethyl brevifolincarboxylate	PHE 66	N…O5	3.55
		ARG 68	NE…O6	3.12
			NH1…O6	3.45
		GLY 69	N…O8	2.89
		GLN 70	O…H31	2.48
		TRP 132	O…H30	2.91
			O…O5	2.78
			N…O5	3.03
		ALA 134	O…H35	2.45
			N…O8	3.03
			N…O7	2.98
4	Quercitrin	PHE 66	O…H43	2.81
			O…H51	2.96
			N…O10	2.83
		PRO 67	O…H41	2.64
		GLY 69	N…O4	3.00
		GLN 70	O…H42	2.98
			O…H41	3.04
		TYR 123	OH…O2	3.53
			OH…O7	2.52
		GLY 124	O…H50	2.96
		ALA 134	N…O4	2.68
5	Curcumin	PHE 66	N…O5	3.21
		GLY 69	N…O4	2.86
		ASN 126	O…H40	2.69
		LYS 127	N…O3	2.56
		ALA 134	N…O4	2.81
6	Ladanein	PRO 67	O…O5	3.30
		ARG 68	NE…O2	2.98
		GLY 69	N…O3	2.89
			N…O4	3.06
		GLN 70	O…H30	2.98
			O…O5	2.44
		ALA 134	O…H31	2.50
			N…O3	2.75
7	Apigenin	ARG 68	NE…O3	3.01
			NH1…O3	3.51
		GLY 69	N…O1	3.25
		GLN 70	O…H29	2.94
		ALA 134	N…O1	3.19
		THR 135	O…H30	3.00
8	Tenofovir	PRO 67	O…N	3.44
		ARG 68	NE…O	3.16
			NE…O	3.10
			NH1…O	2.86
			NH1…O	2.96
		GLN 70	O…N	2.48
			O…N	3.17
		ALA 134	O…N	3.25
			O…N	2.77
9	Resveratrol	GLY 69	N…O2	2.76
		GLN 70	N…O2	2.94
			O…O2H28	2.59
		TYR 123	OH…O1	2.82
		ALA 134	N…O2H28	3.03
10	Ribavirin	PRO 67	O…O5	3.49
		GLY 69	N…O5	2.93
		GLN 70	O…O5	3.07
		TYR 123	OH…N8	2.76
		TRP 132	O…N9	2.66
		ALA 134	N…O5	3.02
			O…O5	3.24
			O…N7	2.81
			O…O2H24	2.68
		GLU 136	OE2…H25	2.85
		GLY 137	N…O4H27	3.28
		ALA 138	N…O4H27	2.98
			O…H27	2.99

SARS-CoV-2, severe acute respiratory syndrome coronavirus 2.
